# The New Era in Teaching

**Published:** 1919-10-04

**Authors:** 


### THE NEW ERA IN TEACHING.

With re-opening of the medical schools we find that the number of students is now greater than ever, and from many points of view this is a most satisfactory situation, particularly when we realise the increasing medical needs of the country, and the fears entertained during the second and third years of the war that we should be faced with a serious insufficiency of doctors. Two main contributory causes may be found. The first is that among the great number of youths entering the Army direct from the public schools without opportunity or need for the moment to select a profession, the survivors' choice of how they shall devote their lives has been noticeably influenced by the generous grants which the Government has offered to ex-Service men who are willing to take up the study of medicine. Also in their war experience these men cannot, in the majority of instances, have failed to encounter striking examples that, whatever may be its financial limitations, the medical profession offers unique opportunities of work which amply repays the effort made. The second cause is largely relative, and rises simply with the return to their studies of young men who have already registered as medical students in 1914-15, but who had not at that time attained a sufficiently advanced stage in their professional training to entitle them to exemption from conscription under the Compulsorily Service Acts.

The result of this situation is that there cannot now be the future dearth of doctors which was at one time forecasted. Also we read that the Irish and Scottish medical schools have contained their fully complement of students throughout almost the whole of the war, and it almost becomes a question of overcrowding the profession. In spite of the fact that the majority of school authorities hold this fear to be probably as groundless as the one-time scare of scarcity, it is a point to be considered when guiding the early steps of the country's youth. Certainly the naval and military services, the Colonies, and public health duties will inevitably be able to absorb more and yet more men as time and development progress, but to those who favour the typical home life of this country and the old style medical practice, this possible surplus and excessive competition combined with the already enormously increased cost of both home and practice, may well call for attention when viewed relatively to the cost of a medical education.

With regard to the training itself, no great and outstanding changes may be obvious at the first glance. The experiences of the war have brought in a number of radical alterations in actual treatment both surgical and medical, and we in this Journal have repeatedly drawn attention to them as the principles involved took shape and crystallised, but most of these matters belong to the later, rather than the early stages of the medical curriculum, and one may probably best describe the post-war teaching changes as reorganisation of the teaching staffs with arrangements whereby their attention shall be much more concentrated upon the actual duties of instruction. The all too common practice of learning but to forget, a scourge in the schools of the past, is to be attacked from all quarters. A process is to be pursued to the utmost of "linking up" training in all branches of scientific knowledge towards one common end, medical care and understanding of mankind. The recent outcry for more medical research will, in most quarters, receive adequate response, but we feel that this development is and will be more the natural result of increased financial public support for such investigational work than a sudden awakening of a slumbering profession. As Sir George Newman stated in his memorandum on Medical Education in England, "the spirit of research has proved itself to be the very breath and being of all true medical progress."

One of the most marked changes has, however, occurred in the student himself. At present, if one can judge by local experience, he is a much more serious and studious individual than his pre-war predecessor. Not that his spirits and joy of life have gone, but rather has he forgotten the traditional medical student of the past who if not already possessed of them, was often as in duty bound to cultivate a fine sense of irresponsibility and complete disregard for the seriousness of life. War has apparently changed a fashion and we think probably to the better advantage of the working of our early medical reforms at least. Possibly the change is but temporary, soon will come a generation who know the war as tales and history only; perhaps they will revert to the type, which most people not unfairly regarded with a very tolerant and sympathetic eye, but we hope that by then we and they will be well launched in the new era of medical teaching.

